# Supplementary material for: Retinal Ganglion Cell Diversity and Subtype Specification from Human Pluripotent Stem Cells
Source: Stem Cell Reports. 2018 Mar 22;10(4):1282–93. doi: 10.1016/j.stemcr.2018.02.010 (PMC5998302; doi:10.1016/j.stemcr.2018.02.010)
Supplement: Document S1. Figures S1 and S2 and Tables S1 and S2 [file mmc1.pdf]

Stem Cell Reports, Volume *10*

Supplemental Information

Retinal Ganglion Cell Diversity and Subtype Specification from Human Pluripotent Stem Cells

Kirstin B. Langer, Sarah K. Ohlemacher, M. Joseph Phillips, Clarisse M. Fligor, Peng Jiang, David M. Gamm, and Jason S. Meyer

**Table S1:** Major RGC Subtype Classes, their molecular makers, and their function		
Retinal Ganglion Cell Subtype	Molecular Markers	Function
ON-OFF Direction Selective-RGCs	CART, CDH6, MMP17, DRD4, HB9, THRH, BD	Respond to preferred directional motion of bright and dark stimuli
ON Direction Selective-RGCs	HOXD10, FSTL4	Respond to preferred directional motion of bright stimulus
Alpha-RGCs	SPP1, KCNG4, CB2, SMI32	Differentially respond to bright and dark stimuli
Intrinsically photosensitive-RGCs	Melanopsin, TBR2	Respond to light to regulate circadian rhythms and pupillary reflex
W3B-RGCs	SDK2, TYW3	Respond to stimuli that fall within the center of the receptive field.
J-RGCs	JAMB	Respond to directional movement of stimuli from soma-to-dendrite
PV-RGCs	PVALB	Transient responses with small receptive fields and strong surrounds.

Table S1: This table indicates the major classes of RGC subtypes, previously identified molecular markers for each class, and their physiological function in the visual transmission. Related to Figures 3-7.

**Table S2**: Primary Antibody Information			
Primary Antibodies	Company	Catalog Number	Dilution
BRN3	Santa Cruz	SC-15375	1:200
βIII-Tubulin	Covance	PRB-435P/802001	1:1000
CART	Phoenix Pharmaceuticals	H-003-62	1:100
CB2	R&D Systems	MAB3655	1:50
CDH6	Sigma-Aldrich	HPA007047	1:100
DCX	Santa Cruz	SC-271390	1:50
FSTL4	Novus	91913	1:200
HUC/D	Molecular Probes	A-21271	1:200
ISLET1	DSHB	40.2D6	1:200
MAP2	Santa Cruz	SC-20172	1:200
MELANOPSIN	ThermoScientific	PA1-781	1:500
SMI-32	Calbiochem Millipore	NE1023	1:500
SPP1	Abcam	Ab91655	1:50

Table S2: Antibodies used in this study, related to Figures 1, 3, 4, 5, and 7.

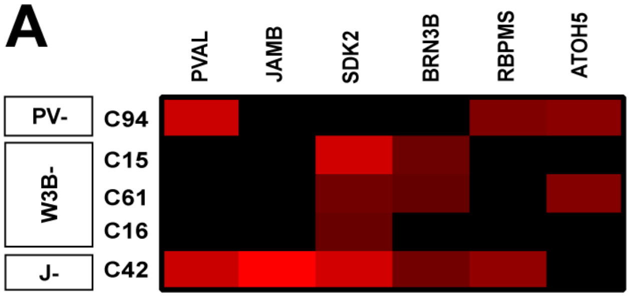

Figure S1: **Single cell qRT-PCR reveals expression of markers for other RGC subtypes.** A variety of other subtypes were characterized using single cell qRT-PCR. (A) PV-, W3B-, and J-RGCs were identified through the expression of PVALB, SDK2, and JAMB respectively, in addition to their combinatorial expression with a variety of other RGC-associated markers.

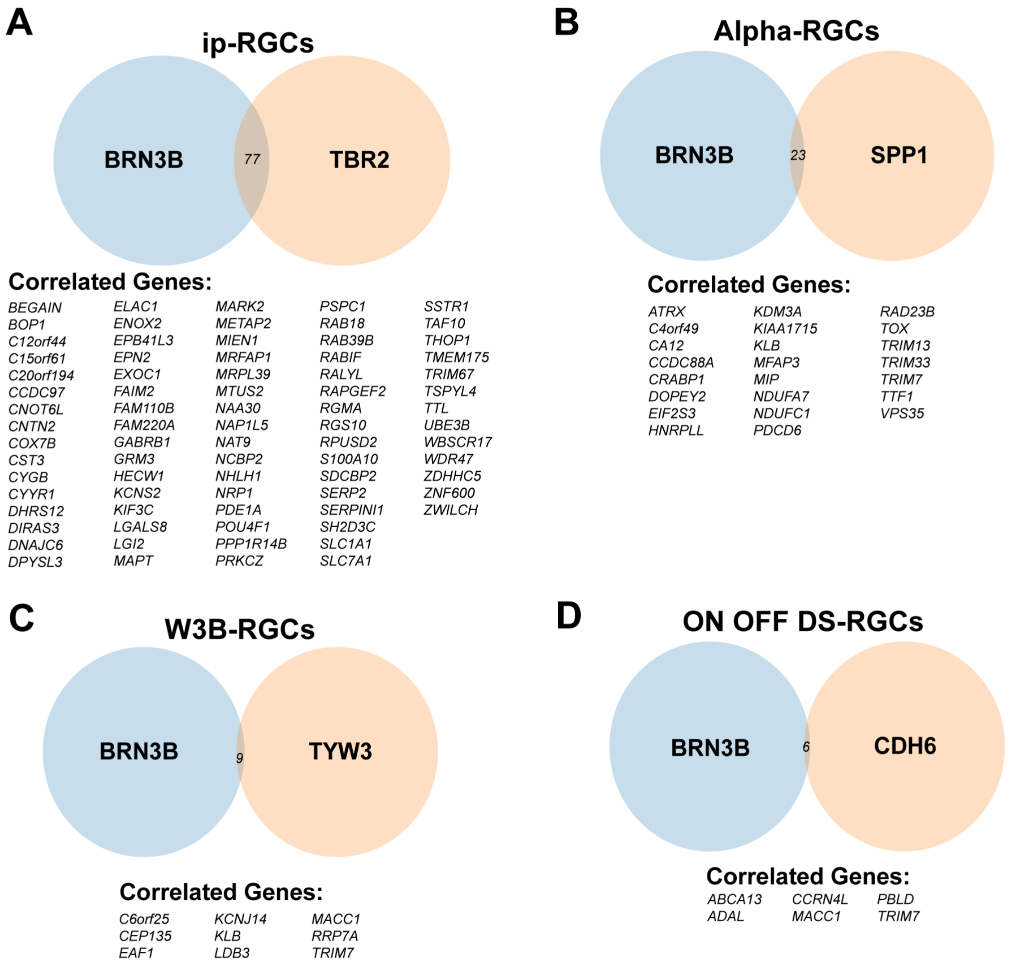

Figure S2: Single cell RNA-seq analyses indicate correlative genes for major RGC subtype classes. Single cell RNAseq analyses of numerous subtypes reveals closely correlated genes. (A-D) SRCCA for BRN3B using the top 1000 correlating genes was combined with SRCCA for subtype specific markers and revealed correlated genes. For ip-, Alpha-, W3B-, and ON OFF DS-RGCs 77, 23, 9, and 6 genes were found closely correlated within the top 1000 genes.
