# Supplementary material for: Retinal Ganglion Cell Diversity and Subtype Specification from Human Pluripotent Stem Cells
Source: Stem Cell Reports. 2018 Mar 22;10(4):1282–93. doi: 10.1016/j.stemcr.2018.02.010 (PMC5998302; doi:10.1016/j.stemcr.2018.02.010)
Supplement: Document S2. Article plus Supplemental Information [file mmc2.pdf]

Stem Cell Reports
Article

OPEN ACCESS

Retinal Ganglion Cell Diversity and Subtype Specification from Human Pluripotent Stem Cells

Kirstin B. Langer,[1] Sarah K. Ohlemacher,[1] M. Joseph Phillips,[2,3] Clarisse M. Fligor,[1] Peng Jiang,[4] David M. Gamm,[2,3,5] and Jason S. Meyer[1,6,7,*]
[1]Department of Biology, Indiana University Purdue University Indianapolis, Indianapolis, IN 46202, USA
[2]Waisman Center, University of Wisconsin-Madison, Madison, WI 53705, USA
[3]McPherson Eye Research Institute, University of Wisconsin-Madison, Madison, WI 53705, USA
[4]Morgridge Institute for Research, Madison, WI 53705, USA
[5]Department of Ophthalmology and Visual Sciences, University of Wisconsin-Madison, Madison, WI 53705, USA
[6]Department of Medical and Molecular Genetics, Indiana University, Indianapolis, IN 46202, USA
[7]Stark Neurosciences Research Institute, Indiana University, Indianapolis, IN 46202, USA
*Correspondence: meyerjas@iupui.edu
https://doi.org/10.1016/j.stemcr.2018.02.010

SUMMARY

Retinal ganglion cells (RGCs) are the projection neurons of the retina and transmit visual information to postsynaptic targets in the brain. While this function is shared among nearly all RGCs, this class of cell is remarkably diverse, comprised of multiple subtypes. Previous efforts have identified numerous RGC subtypes in animal models, but less attention has been paid to human RGCs. Thus, efforts of this study examined the diversity of RGCs differentiated from human pluripotent stem cells (hPSCs) and characterized defined subtypes through the expression of subtype-specific markers. Further investigation of these subtypes was achieved using single-cell transcriptomics, confirming the combinatorial expression of molecular markers associated with these subtypes, and also provided insight into more subtype-specific markers. Thus, the results of this study describe the derivation of RGC subtypes from hPSCs and will support the future exploration of phenotypic and functional diversity within human RGCs.

INTRODUCTION

Retinal ganglion cells (RGCs) are the projection neurons of the visual system responsible for transmission of signals from the retina to the brain (Levin, 2005; Rokicki et al., 2007). While long-distance connectivity with postsynaptic targets is common to all RGCs, these cells may differ in their physiological roles exhibiting varied responses to visual stimuli. Traditionally, these functional differences among RGCs have been characterized by physiological parameters as well as variation in their dendritic arborization within the inner plexiform layer (Berson, 2008; Dhande et al., 2015; Jacoby and Schwartz, 2017; Sanes and Masland, 2015; Sümbül et al., 2014; Struebing et al., 2016). More recently, a number of molecular markers have been described to further classify different subtypes of RGCs, with more than 30 different subtypes identified to date (Huberman et al., 2009; Kay et al., 2011; Kim et al., 2008; Rousso et al., 2016; Sanes and Masland, 2015; Siegert et al., 2012; Sweeney et al., 2017).

Many previous studies focused upon RGC subtypes have relied upon the use of animal models, leading to the ability to identify these cells and study their functional characteristics (Dhande et al., 2015; Sanes and Masland, 2015). However, the study of RGC subtypes in the human system has been lacking, due to the limited availability of adult tissue and the inaccessibility of the human retina at early developmental stages. Human pluripotent stem cells (hPSCs) provide a powerful tool for studies of cell-type diversity, as they have the ability to self-renew and give rise to all cell types of the body (Takahashi et al., 2007; Trimarchi et al., 2007, 2008; Yu et al., 2007). Many previous efforts have examined the ability of hPSCs to give rise to RGCs (Gill et al., 2016; Ohlemacher et al., 2016; Riazifar et al., 2014; Sluch et al., 2015; Tanaka et al., 2016; Teotia et al., 2017). However, this differentiation has focused upon the generation of RGCs as a whole, without a focus on the numerous subtypes that are known to exist. The ability to generate these cells from hPSCs allows for the study of the cellular mosaicism that exists among RGCs of the human retina, with important implications for how these cells differ in their function as well as how they may be affected in disease states.

To address these shortcomings, efforts of the current study were focused upon the identification of multiple RGC subtypes derived from hPSCs. Numerous RGC subtypes were identified within cultures of hPSC-derived RGCs based upon their expression of characteristic genes, including the combinatorial expression of molecular markers allowing for their classification into a proper subtype class. The results of this study explored the identification of RGC subtypes within hPSC-derived retinal cultures. This study also provides the foundation for a more comprehensive analysis of hPSC-derived RGCs in future studies, including the investigation of important functional and physiological differences between RGC subtypes.

© 2018 The Author(s).
This is an open access article under the CC BY license (http://creativecommons.org/licenses/by/4.0/).

RESULTS

Cellular Diversity of RGCs Derived from hPSCs

RGCs serve as the functional connection between the eye and the brain, extending lengthy axons vital for the transmission of visual information (Levin, 2005; Rokicki et al., 2007). As such, the ability to derive RGCs from hPSCs has been an increasingly important field of research in recent years, with the potential to use these cells not only for cellular replacement, but also for their use in disease modeling and pharmacological screening. Previous studies that have investigated the derivation of retinal cells from hPSCs have largely focused upon these populations as a whole (Hirami et al., 2009; Lamba et al., 2006; Meyer et al., 2011; Meyer et al., 2009; Ohlemacher et al., 2015, 2016; Osakada et al., 2009; Sridhar et al., 2013, 2016; Zhong et al., 2014), with the identification of RGCs relying solely upon the expression of BRN3, a transcription factor specific to such cells in the retina. However, as the projection neurons of the retina, RGCs exhibit numerous morphological, phenotypic, and functional differences associated with the roles they play in visual transduction. Thus, initial efforts were undertaken to examine hPSC-derived RGC populations for differences in their transcriptional profiles to identify genes that may constitute a core network of genes associated with a variety of RGC subtypes, as well as those that may be expressed in subsets of RGCs.

hPSC-derived RGCs exhibited elaborate morphological features including the extension of lengthy neurites within the first 80 days of differentiation (Figure 1). These RGCs exhibited numerous characteristic features, with large three-dimensional somas interconnected by long neuronal processes (Figure 1A). The RGC fate of these cells was confirmed by the expression of RGC-associated proteins, including transcription factors such as BRN3, ISL1, and SNCG, as well as long and interconnecting neurites expressing a variety of cytoskeletal markers (Figures 1B–1D). More so, the diversity among RGCs was demonstrated by co-staining four of the most common RGC-associated markers and indicated a variation in expression levels throughout the RGC population (Figures 1E–1J). The resultant RGCs were found to express a complete profile of RGC-associated features and demonstrated a unique diversity in RGC-associated gene expression.

The transcriptional profiles of these hPSC-derived RGCs were analyzed by single-cell RNA sequencing (RNA-seq), with numerous genes found to be expressed in all RGCs, while others were expressed within subsets of the complete RGC population (Figure 2). Spearman's rank correlation coefficient analysis (SRCCA), a technique used to find genes that correlate most closely with one or more designated target genes across the entirety of a single-cell RNA-seq dataset (Phillips et al., 2017), was used to elucidate single-cell data. Commonly studied genes associated with RGCs were used as target genes and SRCCA was performed to identify those genes whose expression was strongly correlated with the expression of these targets across the entire dataset (Figures 2A–2C). Results of these analyses revealed evident clustering of expression patterns within RGCs.

More so, SRCCA correlations from multiple target genes can be combined to identify genes specific to a given cell type. To identify unique RGC markers, SRCCA identified the 200 genes most strongly correlating with *BRN3B*, *ISL1*, *SNCG*, and *RBPMS*. This analysis revealed 11 genes strongly correlated with each of the 4 target genes (Figure 2D), including several with known roles predominantly in neuronal cell types. This analysis not only revealed numerous similarities in RGC gene expression but also indicated a distinct heterogeneity within this population of cells in which numerous genes were found to be correlated with only some of the target genes. Thus, these differences were explored to identify RGC subtypes within the hPSC-derived RGC population.

Identification of Defined RGC Subtypes within hPSC-Derived Cultures

Although some previous studies have demonstrated the ability of hPSCs to give rise to RGCs (Gill et al., 2016; Ohlemacher et al., 2016; Riazifar et al., 2014; Sluch et al., 2015; Tanaka et al., 2016; Teotia et al., 2017), the diversity of subtypes among RGCs has been largely overlooked. More than 30 different subtypes of RGCs are known to exist, varying in their gene expression patterns, morphological features, and functionality (Dhande et al., 2015; Sanes and Masland, 2015). Given the variability in gene expression observed among hPSC-derived RGCs (Figure 2), efforts were undertaken to further examine the specific RGC subtypes present within these cultures based on the expression of genes characteristic for each class (Table S1). Direction-selective RGCs (DS-RGCs) constitute a group of cells capable of responding to preferred directional motion in response to bright and dark stimuli, with the identification and functionality of these cells previously explored in animal models (Cruz-Martín et al., 2014; Huberman et al., 2009; Kay et al., 2011; Sanes and Masland, 2015; Wei and Feller, 2011). However, such cells have not been definitively identified in human RGCs. DS-RGCs have been divided into two groups: ON-OFF DS-RGCs capable of detecting preferred directional motion to bright and dark stimuli, and ON DS-RGCs which respond to the directional motion of a bright stimulus. Each category of DS-RGC has been identified by the expression of specific molecular markers, such as CART, CDH6, and FSTL4, and further characterized into subgroups based upon preferred directional motions (dorsal, ventral, nasal, and temporal)

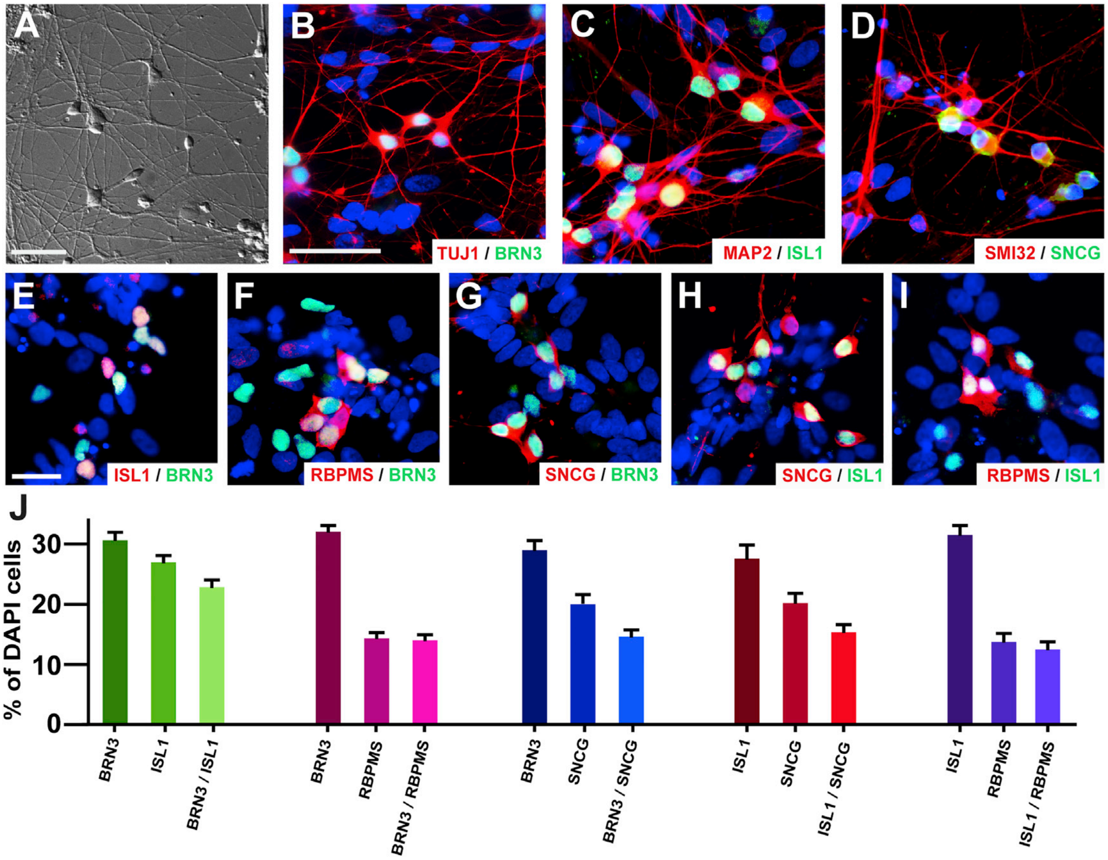

Figure 1. hPSC-Derived RGCs Display Elaborate Morphologies and Diversity of Gene Expression

(A) Differential interference contrast (DIC) imaging demonstrated morphological characteristics of hPSC-derived RGCs with large three-dimensional cell bodies, projecting numerous lengthy neurites.

(B–D) Immunocytochemistry confirmed the RGC identity of these cells through the expression of specific markers such as (B) BRN3, (C) ISL1, and (D) SNCG, as well as the extension of long processes indicated by cytoskeletal markers.

(E–I) In addition, analysis of RGC markers revealed varying degrees of co-expression within the RGC population, (E) ISL1 and BRN3, (F) RBPMS and BRN3, (G) SNCG and BRN3, (H) SCNG and ISL1, and (I) RBPMS and ISL1.

(J) Quantification of immunocytochemistry results verified variation in RGC-associated gene expression among hPSC-derived RGCs. Scale bars, 50 μm in (A)–(D), 25 μm in (E)–(I); the scale bar in (B) applies to (B)–(D); the scale bar in (E) applies to (E)–(I). Error bars represent SEM (n = 27 technical replicates from 3 biological replicates for each bar using miPS2, H9, and H7 cell lines).

through the combinatorial expression of specific genes (De la Huerta et al., 2012; Huberman et al., 2009; Kay et al., 2011; Yonehara et al., 2008).

DS-RGC-like cells could be identified in hPSC cultures within the first 80 days of differentiation, with the expression of BRN3 serving as confirmation of their RGC lineage. Presumptive ON-OFF DS-RGCs were identified based upon their expression of CART in 25.95% ± 0.46% of the BRN3-positive RGC population (Figures 3A and 3D). DS-RGCs can also be further divided into ON-OFF as well as ON subgroups, identifiable based on the expression of genes such as CDH6 and FSTL4, respectively. Thus, a more detailed analysis of hPSC-derived RGCs revealed that CDH6-positive ON-OFF DS-RGC-like cells were identified within 17.16% ± 0.51% of the BRN3-positive RGC population (Figures 3B and 3D). Similarly, the expression of FSTL4, indicative of ON DS-RGCs, was found within 30.49% ± 0.58% of BRN3-positive RGCs (Figures 3C and 3D). As the combinatorial expression of several genes is often required for the identification of some RGC subtypes, single-cell qRT-PCR confirmed the combined expression of numerous DS-RGC markers within individual RGCs (Figure 3E).

α-RGCs represent another RGC subtype which vary in their response to bright and dark stimuli with their responses, including transient OFF, sustained OFF, and ON (Dhande et al., 2015; Krieger et al., 2017). Previous studies have demonstrated high levels of SPP1 expressed in α-RGCs, with this gene currently serving as the main identifier of this class of cells (Duan et al., 2015; Pang et al.,

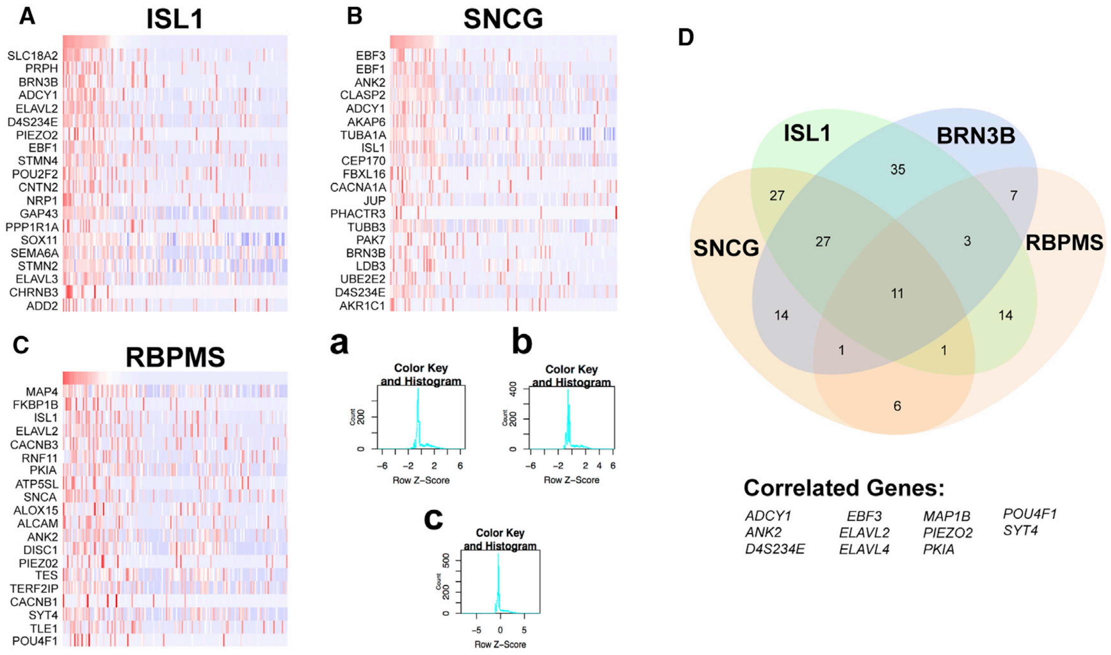

Figure 2. hPSC-Derived RGCs Demonstrate Unique Transcriptional Profiles

(A–C) Spearman's rank correlation coefficient analysis (SRCCA) was performed on single-cell RNA-seq data using RGC-specific target genes, (A) ISL1 (B) SNCG and (C) RBPMS. Cells were ranked from highest to lowest expression and transformed into *Z* scores. Results were constructed into a heatmap, with the top 20 correlating genes shown for each RGC marker. Corresponding color key histograms for (A)–(C) are displayed in a–c.

(D) The combination of SRCCA from four RGC target genes for the top 200 correlating genes revealed differential gene expression as well as a core set of 11 genes highly expressed within RGCs. n = 3 biological replicates using the H9 cell line.

2003). Additional markers such as KCNG4 and CB2 have also been identified to further classify α-RGCs (Duan et al., 2015; Huberman et al., 2008). Thus, the presence of α-RGCs within the hPSC-derived RGC population was investigated using these genetic markers.

Presumptive α-RGCs were identified by the combinatorial expression of α-RGC-associated genes in conjunction with the pan-RGC marker, BRN3. SPP1 expression was discovered in 21.10% ± 0.42% of the BRN3-positive RGCs (Figures 4A and 4C). Further analysis demonstrated the expression of CB2, which identifies a subset of α-RGCs with the capability of having a transient response to dark stimulus, co-localized in 15.72% ± 0.45% of BRN3-RGCs (Figures 4B and 4C). More so, single-cell qRT-PCR analyses confirmed the combinatorial expression of SPP1 and KCNG4 within individual RGCs, characteristic of α-RGCs, in addition to the expression of a variety of other RGC-associated markers (Figure 4D).

Intrinsically photosensitive RGCs (ipRGCs) constitute an additional RGC subtype, with the unique ability to directly respond to light stimuli with the photopigment OPN4 (Hattar et al., 2003; Schmidt et al., 2011). Various subtypes of ipRGCs have been shown to control aspects of circadian rhythm as well as pupillary reflexes by projecting to various non-image processing areas in the brain (Chew et al., 2017; Panda et al., 2002; Sweeney et al., 2014). Previous studies have discovered ipRGC subcategories (M1–M5), which vary in their genetic signature, light response, and physiological functions, although studies of human ipRGCs have been more limited (Ohlemacher et al., 2016; Sluch et al., 2015). As such, a detailed analysis was performed to explore the presence and transcriptional profile of ipRGC-like cells within the hPSC-derived RGC population.

OPN4 was expressed either with or without the co-expression of BRN3, consistent with previous studies that have demonstrated BRN3 expression confined to only some types of ipRGCs (Schmidt et al., 2011) (Figures 5A and 5B). This OPN4 expression was observed in 1.17% ± 0.06% of all cells in conjunction with BRN3, while 2.75% ± 0.13% of all differentiated retinal neurons expressed OPN4 in the absence of BRN3 (Figure 5C). Single-cell transcriptional analyses verified the expression of OPN4 within individual RGCs in combination with BRN3, as well as with the combinatorial expression of other RGC-related markers (Figure 5D).

Furthermore, a variety of other RGC subtypes have been previously identified including PV-, W3B-, and J-RGCs (Goodman et al., 2016; Kim et al., 2008; Kim and Jeon,

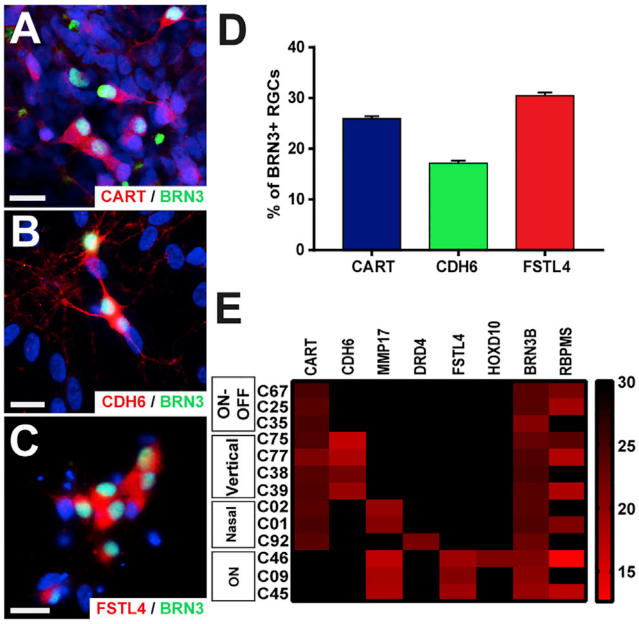

Figure 3. hPSCs Give Rise to Multiple DS-RGC Subtypes
(A and B) ON-OFF DS-RGCs were identified by the co-expression of the RGC marker BRN3 with either (A) CART or (B) CDH6.
(C) ON DS-RGCs were identified by the expression of FSTL4 co-localized with BRN3.
(D) Quantification of immunocytochemistry results identified the expression of CART, CDH6, and FSTL4 within the BRN3-RGC population at 25.95% ± 0.46%, 17.16% ± 0.51%, and 30.49% ± 0.58%, respectively.
(E) Single-cell qRT-PCR analysis demonstrated the combinatorial expression of DS-RGC markers, in conjunction with RGC-associated markers. Error bars represent the SEM (n = 36 technical replicates from 3 biological replicates for each bar using miPS2, H9, and H7 cell lines).
Scale bars, 20 μm.

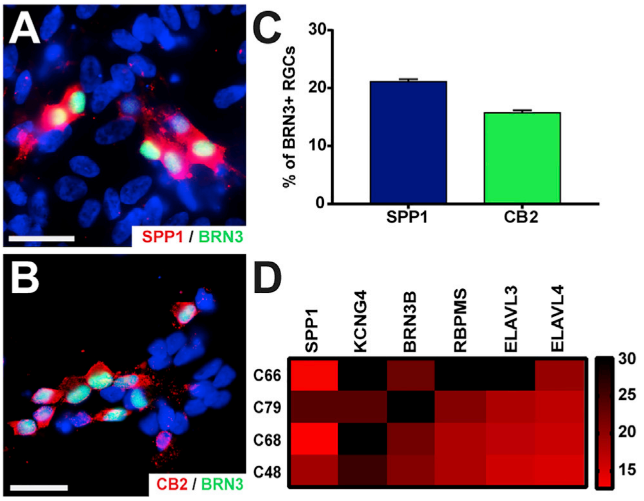

Figure 4. Identification of α-RGCs in hPSC-Derived RGCs
(A and B) α-RGCs were identified by the co-localization of BRN3 with either (A) SPP1 or (B) CB2.
(C) Quantification of immunocytochemistry results indicated SPP1 and CB2 were co-expressed with BRN3 in 21.10% ± 0.42% and 15.72% ± 0.45% of all cells, respectively.
(D) Single-cell qRT-PCR analyses demonstrated the combinatorial expression of α-RGC markers, along with the expression of pan-RGC markers. Error bars represent the SEM (n = 36 technical replicates from 3 biological replicates for each bar using miPS2, H9, and H7 cell lines).
Scale bars, 30 μm.

2006; Krishnaswamy et al., 2015). Single-cell qRT-PCR analyses demonstrated the expression of parvalbumin, SDK2, and JAM-B, which indicated the presence of such subtypes within hPSC-derived cultures, respectively (Figure S1).

Investigation of Additional Molecular Markers for DS-RGCs

The ability to reliably identify specific RGC subtypes based on the expression of molecular markers has only recently been described (Dhande et al., 2015; Sanes and Masland, 2015), with a relatively limited set of markers associated with these subtypes (Table S1). The investigation of additional molecular markers strongly correlated with these subtypes would not only provide for a greater ability to identify specific types of cells, but could also provide some insight into differences in functionality between these cells. SRCCA approaches provide the power to elucidate transcriptional differences between individual cell types and, as such, provide an advantageous tool for the identification of molecular markers associated with individual RGC subtypes. As DS-RGCs were among the most prevalent subtypes identified within cultures of hPSC-derived RGCs (Figure 3C), efforts were made to identify those genes that were most closely correlated with this phenotype. Additional analyses were also performed to identify those genes most strongly correlated with markers of additional RGC subtypes (Figure S2).

For analysis of DS-RGCs, SRCCA was performed to identify those genes that were most strongly correlated with the expression of *FSTL4*, *BRN3B*, and *SNCG*, with numerous genes strongly correlated with the three target genes (Figure 6A). Furthermore, combined SRCCA was performed with *FSTL4* and genetic markers for retinal progenitors, RPE, and photoreceptors. Overlap between *FSTL4* and markers for each of these latter cell types was minimal, indicating a strong degree of specificity for *FSTL4* expression in RGCs (Figures 6B–6D). The results of this analysis provided a total of 148 genes that could serve as genetic identifiers for DS-RGCs. Of these genes, *DCX* was further explored. Previous studies have identified a role for DCX in the early neurogenesis of the CNS; however, its pattern of expression in the retina has not been studied in great detail with its

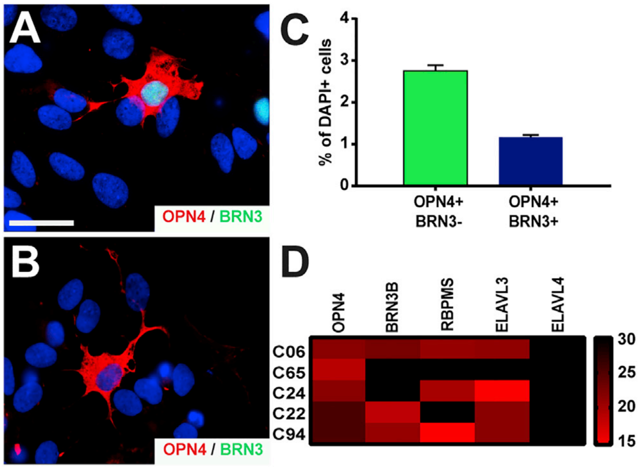

Figure 5. Characterization of Intrinsically Photosensitive RGCs Derived from hPSCs

(A and B) A subset of hPSC-derived cells exhibited the expression of melanopsin co-expressing either (A) with or (B) without BRN3.

(C) Quantification of immunocytochemistry results was performed as a percentage of the total DAPI-positive population and melanopsin-positive/BRN3-negative cells comprised 2.75% ± 0.13% of all cells, while melanopsin-positive/BRN3-positive comprised 1.17% ± 0.06% of all cells.

(D) Single-cell qRT-PCR revealed the combinatorial expression of melanopsin with a variety of other RGC-related markers. Error bars represent the SEM (n = 36 technical replicates from 3 biological replicates for each bar using miPS2, H9, and H7 cell lines).

Scale bar, 50 μm.

expression found in the RGC layer in only a small number of studies (Gleeson et al., 1999; Rachel et al., 2002; Trost et al., 2014). Therefore, the association of DCX with a specific subtype of RGC, namely DS-RGCs, was further investigated in hPSC-derived cells. Immunocytochemistry results revealed DCX expression highly co-expressed with DS-RGC markers such as FSTL4 (Figure 7A), but only in a subset of BRN3- and SNCG-expressing RGCs (Figures 7B and 7C). BRN3-expressing RGCs co-immunostained for DCX in 42.61% ± 1.88% of the population and SNCG-positive RGCs expressed DCX in 53.57% ± 1.88% of the RGCs. More so, quantification revealed that FSTL4-positive RGCs co-localized with DCX at 82.48% ± 1.66% (Figure 7D). In addition, single-cell RNA-seq demonstrated the specificity of DCX expression with DS-RGCs apart from other RGCs and retinal cell types (Figure 7E). Thus, the results of this analysis have identified DCX as a potentially useful marker for DS-RGCs.

DISCUSSION

The ability to derive RGCs from hPSCs has been the subject of several recent studies, as these cells function to transmit visual information between the eye and the brain, and are functionally compromised in several blinding disorders (Levin, 2005; Rokicki et al., 2007). However, these studies have investigated RGCs as a generic population (Gill et al., 2016; Ohlemacher et al., 2016; Riazifar et al., 2014; Tanaka et al., 2016; Teotia et al., 2017), with little emphasis upon the diversity of RGCs known to exist. To date, numerous RGC subtypes have been identified within animal models based upon morphological features as well as functional properties (Dhande et al., 2015; Sanes and Masland, 2015), although such subtypes within human RGCs have been much less characterized. Serving as the projection neuron of the retina, RGC subtypes vary in their functional properties and work cohesively in order to properly transmit visual information to the brain (Huberman et al., 2008). The generation of hPSC-derived RGCs in a manner that represents the diversity of RGC subtypes provides a more reliable and realistic model for studying the development of RGCs, with important implications on how individual subtypes may be differentially affected in various disease states. The recent discovery of molecular markers for specific RGC subtypes facilitates the ability to identify these populations within hPSC-derived RGCs (Dhande et al., 2015; Sanes and Masland, 2015). The results of this study represent efforts to extensively investigate and identify RGC subtypes within hPSC-derived retinal cells. Furthermore, these studies also contribute to a greater characterization of human RGC subtypes, with results identifying additional molecular markers for major classes of these cells.

RGCs exhibit numerous unique characteristics that set them apart from other retinal cell types, such as their morphological appearance and their transcriptional profile. The expression of many genes can be used to identify RGCs, although the expression of BRN3B, ISL1, SNCG, and RBPMS have been among the most widely utilized molecular markers. Each of these markers was observed within populations of hPSC-derived RGCs, although RBPMS was observed less frequently than the other RGC markers. Traditionally, RBPMS is considered to be a pan-RGC marker, therefore the reduced expression of this gene is a likely indicator that RBPMS is expressed somewhat later than the other RGC markers analyzed. Thus, when studying early stages of RGC development with hPSCs, the use of RBPMS likely underestimates the total RGC population.

SRCCA analysis was used in the current study to confirm the expression of a number of other RGC-associated genes that were highly correlated with these four commonly utilized RGC markers. Interestingly, however, the most highly correlated genes associated with each of these four markers revealed striking differences in the transcriptional profiles of these cells, suggesting diversity among the RGC population. In addition, the diversity among RGCs became more

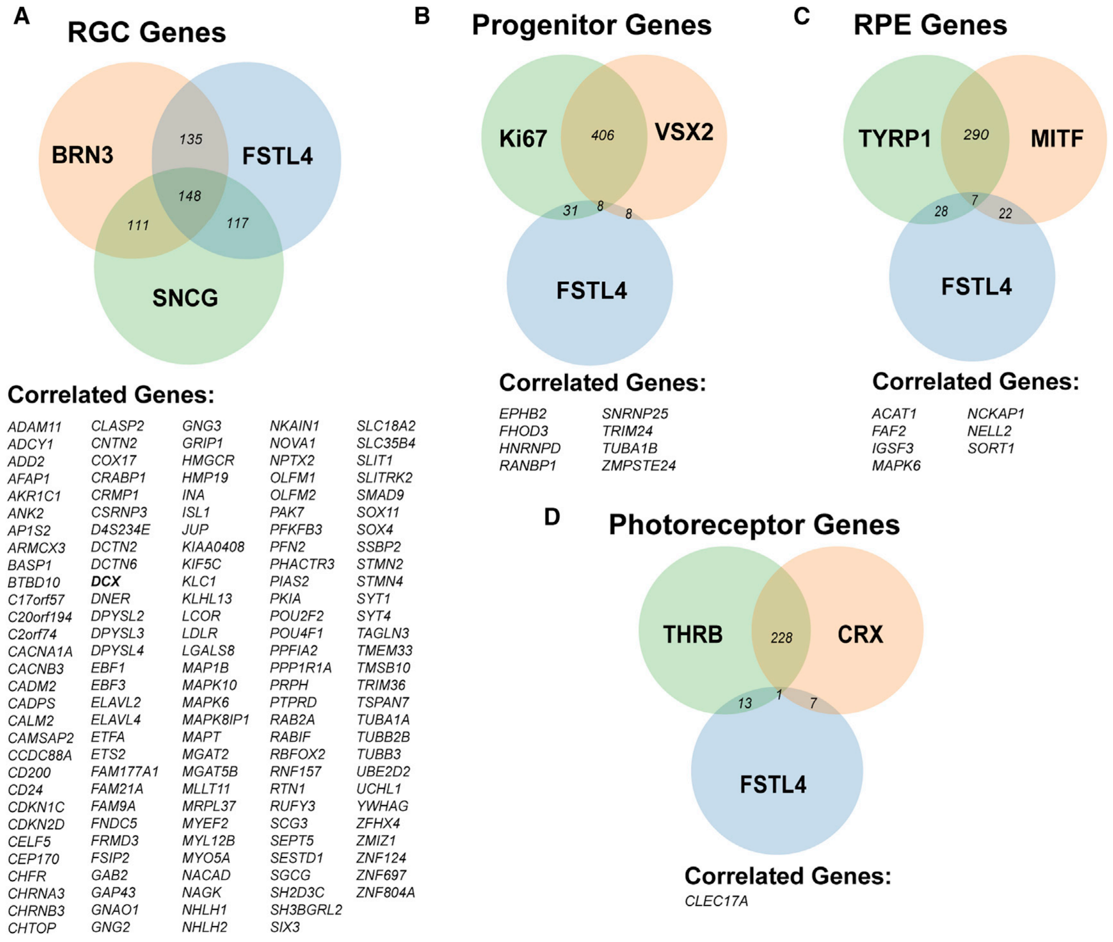

Figure 6. Identification of DS-Associated Markers Using Single-Cell RNA-Seq Analysis

(A) SRCCA from *FSTL4*, *BRN3B*, and *SNCG* were combined for the top 1,000 correlating genes, and 148 genes were found to be commonly expressed between the 3 populations.

(B–D) In addition, SRCCA for *FSTL4* was combined with (B) retinal progenitor genes, (C) RPE genes, and (D) photoreceptor genes and demonstrated minimal overlapping expression. n = 3 biological replicates using the H9 cell line.

apparent when SRCCA analysis from the four target genes was combined for the top 200 genes correlating with all of these markers. These results displayed heterogeneity within RGCs through the differential expression of correlating markers, and this diversity among RGCs could potentially be explained by the presence of numerous subtypes within the RGC population. Thus, the results displayed by single-cell RNA-seq analysis prompted the exploration of RGC subtypes within hPSC-derived cells.

The varied subtypes of RGCs have received greater attention in recent years, as they each play important but functionally distinct roles in the visual transduction pathway (Dhande et al., 2015). Traditionally, efforts have relied largely on dendritic morphology to identify and characterize RGC subtypes, with different subtypes encompassing unique sizes, shapes, and arborization within the inner plexiform layer (Dhande et al., 2015; Sanes and Masland, 2015; Triplett et al., 2014). For example, many primate RGCs have often been clustered into categories including midget RGCs or parasol RGCs based upon such morphological features (Croner and Kaplan, 1995; Kaplan and Benardete, 2001; Leventhal et al., 1981). Such studies have been more complicated for human RGC subtypes, due to a more limited access to the retina for experimental investigation. More recently, the development of molecular markers associated with specific subtypes in rodent models has facilitated the ability to identify these cells more readily (Sanes and Masland, 2015). However, such molecular markers remain largely unexplored for primate RGCs, including humans (Rousso et al., 2016). The results of this study show that molecular markers for major subtype classes could be utilized to readily identify RGC subtypes in hPSC-derived RGCs, representing exciting new avenues for research in RGC diversity. In addition, the opportunity exists to study

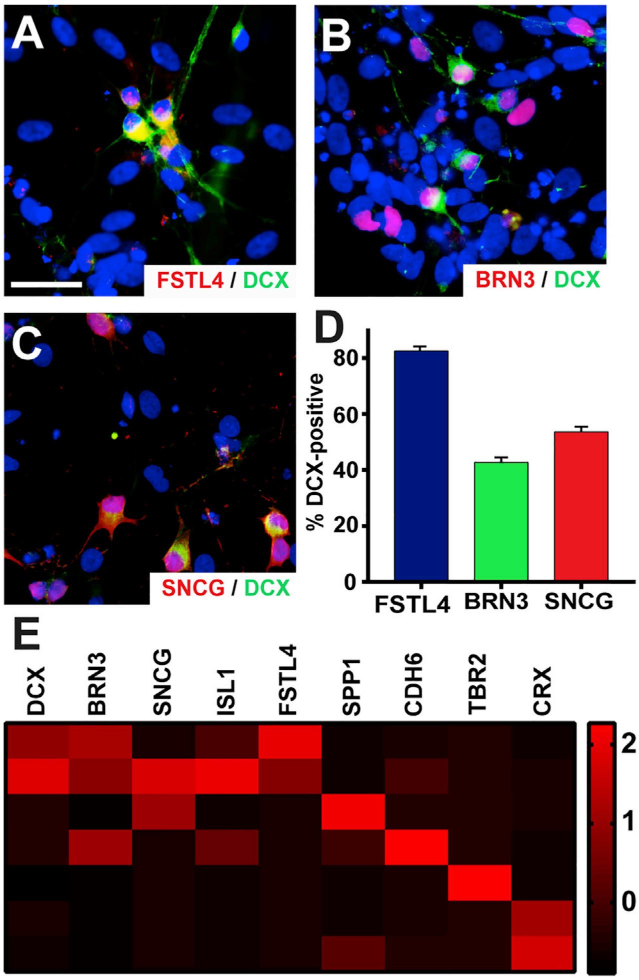

Figure 7. Identification and Confirmation of DCX as a DS-RGC Marker

(A–C) DCX was highly co-localized with (A) FSTL4, while its co-expression with pan-RGC markers (B) BRN3 and (C) SNCG demonstrated less correlation.

(D) Quantification of immunocytochemistry results indicated that DCX expression correlated with 82.48% ± 1.66% of FSTL4-positive RGCs, while it was identified in subsets of BRN3- and SNCG-positive RGCs at 42.61% ± 1.88% and 53.57% ± 1.88%, respectively.

(E) Single-cell RNA-seq values demonstrate expression of DCX correlated with other DS-RGC markers, but was found exclusive of markers of other RGC subtypes and retinal cells.

Scale bars, 50 μm. Error bars represent SEM (n = 30 technical replicates from 3 biological replicates for each bar using miPS2, H9, and H7 cell lines).

species-specific differences in the expression of molecular markers corresponding to RGC subtypes. In the current study, some molecular markers that have been used to identify mouse RGC subtypes (for example, HB9 and HoxD10) were not found to be expressed in hPSC-derived RGCs. While this difference could possibly be due to a degree of maturation, it is also possible that these proteins do not serve as effective markers for RGC subtypes in human cells, highlighting the importance of examining these molecular markers among many different species.

Furthermore, a more enhanced characterization of RGC subtypes was performed in the current study through the analysis of single-cell transcriptional profiles, with this exploration providing the ability to identify molecular markers of RGC subtypes, particularly those for human RGCs. In combination with the RGC-specific marker BRN3B, SRCCA analyses performed with multiple RGC subtype-specific genes revealed identifiers for major subtype classes. In particular, the combination of *BRN3B* and *SNCG* with the direction selective-RGC marker *FSTL4* demonstrated a high number of similarly correlated genes, as opposed to a very limited number of correlated genes when FSTL4 was paired with molecular markers of other retinal cell types. As such, this list of genes highly correlated with the expression of *BRN3B*, *SNCG*, and *FSTL4* could provide important information about the functionality of these cells, as well as provide candidates to serve as molecular markers. Among these highly correlated genes, *DCX* was identified as an intriguing candidate within the list of genes highly correlated with both *FSTL4* and *BRN3B*. Previous work has demonstrated a role for DCX in neurogenesis in the CNS, and its expression has also been observed within the RGC layer in a limited number of studies (Gleeson et al., 1999; Rachel et al., 2002; Trost et al., 2014). In the current study, DCX expression was found to be exclusively expressed within RGCs, although only a small subset of RGCs expressed DCX, indicating the specificity of DCX expression within a given RGC subtype. In addition, a high degree of correlation was observed between DCX- and FSTL4-positive DS-RGCs, demonstrating the possible utility of DCX as a molecular marker for DS-RGCs. Thus, the observed expression of DCX is likely indicative of a particular subset of RGCs, rather than an indicator of their degree of maturation. These results highlight the use of SRCCA for the discovery and validation of additional genes expressed within specific RGC subtypes.

The identification of RGC subtypes using molecular markers can also reveal numerous aspects of RGC development that have not been explored to date. The use of hPSCs as a model system provides a powerful tool to study some of the earliest events in human retinal development (Keller, 2005). The identification of RGC subtypes using molecular markers early in retinal development can also uncover important signaling pathways that could lead to the differentiation of one subtype over another, with varying subtypes requiring different signaling cues for proper development. Furthermore, as each subtype varies in their functional characteristics, more detailed analyses of

individual subtypes allows a greater understanding of their specific roles in the visual transduction pathway.

In addition, blinding diseases which cause degeneration and eventual death of RGCs often appear to preferentially target one subtype over others (Duan et al., 2015; El-Danaf and Huberman, 2015; Majander et al., 2017; Ohlemacher et al., 2016; Puyang et al., 2017). With the advancement of patient-specific hPSCs, RGC subtypes can be identified using molecular markers in disease models such as glaucoma, with an emphasis on whether one subtype is predominantly targeted for degenerative effects compared with other subtypes. The ability to investigate the effects of degenerative diseases on specific RGC subtypes allows for a more refined and targeted approach to eventual treatments for optic neuropathies, including the development of pharmaceutical and cell replacement strategies to counter the degenerative processes observed in each subtype.

EXPERIMENTAL PROCEDURES

Maintenance of hPSCs

Human pluripotent stem cell lines miPS2, H7, and H9 (Sridhar et al., 2016; Thomson et al., 1998) were grown and maintained in their pluripotent state following previously described protocols (Meyer et al., 2009, 2011; Ohlemacher et al., 2015). In brief, hPSCs were maintained upon Matrigel-coated six-well plates in mTeSR1 medium (STEMCELL Technologies). Cells were passaged upon reaching approximately 70% confluency. Undifferentiated cells were examined before passaging for areas of spontaneous differentiation, and such areas were marked and mechanically removed. Colonies were then enzymatically lifted using dispase for approximately 15 min, and hPSCs were then replated at a 1:6 ratio.

Differentiation of hPSCs

Retinal differentiation of hPSCs was accomplished with modifications to previously described protocols (Meyer et al., 2009, 2011; Ohlemacher et al., 2015). Differentiation was initiated via the formation of embryoid bodies, which were then transitioned over a 3-day period from mTeSR1 medium to neural induction medium ([NIM]: DMEM/F12 [1:1], N2 supplement, minimal essential medium [MEM] nonessential amino acids, heparin [2 mg/mL], and penicillin-streptomycin). After a total of 7 days of differentiation, embryoid bodies were induced to adhere to a six-well plate using 10% fetal bovine serum (FBS) in NIM overnight. The next day, FBS was removed and cells were grown in NIM until day 16, with a media change every other day. At this time point, colonies were mechanically lifted and transferred into suspension culture as cell aggregates in retinal differentiation medium ([RDM]: DMEM/F12 [3:1], MEM nonessential amino acids, B27 supplement, and penicillin-streptomycin). Suspension cultures yielded the formation of both retinal organoids and non-retinal forebrain neurospheres, with the retinal organoids identified and isolated based on their morphological characteristics within 30 days of differentiation. After a total of 40 days of differentiation, retinal organoids were dissociated into a single-cell suspension using Accutase and plated on coverslips at a concentration of 50,000 cells per 12-mm coverslip. Cells were maintained up to 80 days of differentiation using BrainPhys Neuronal Medium, with a media change twice a week.

Immunocytochemistry

Cultures of RGCs at 80 days of differentiation were fixed with 4% paraformaldehyde in PBS for 30 min at room temperature. Samples were then washed three times with PBS, followed by permeabilization using 0.2% Triton X-100 solution for 10 min. Cells were then blocked with 10% donkey serum at room temperature for 1 hr. Primary antibodies (Table S2) were diluted in 5% donkey serum and 0.1% Triton X-100 and applied to cells overnight at 4°C. The following day, cells were washed three times with PBS and blocked with 10% donkey serum for 10 min. Secondary antibodies were diluted at a concentration of 1:1,000 in 5% donkey serum and 0.1% Triton X-100 and added to cells for 1 hr at room temperature. Cells were then washed three times with PBS before mounting onto slides for microscopy.

Microscopy and Data Quantification

Following immunostaining, RGCs were imaged using a Leica DM5500 fluorescence microscope. Four regions from multiple coverslips were imaged for the expression of the RGC marker BRN3 and various subtype markers, and these experiments were replicated three times using separately differentiated batches of cells. The number of RGCs were quantified based upon BRN3 expression using ImageJ cell counter plugin, and subtype markers were quantified according to their co-expression with BRN3. The average number of cells expressing each subtype marker in conjunction with BRN3 was quantified along with the SEM were determined using GraphPad Prism software.

Single-Cell qRT-PCR Data Collection and Analysis

Within 80 days of differentiation, hPSC-derived RGCs were dissociated with Accutase for 10 min. Single cells were sorted using the SORP Aria cell sorting machine. A total of 10,000 cells were loaded into capture sites of the C1 Single-Cell Auto Prep fluidic circuit. After loading, individual capture sites from the C1 plate were examined and the contents of each capture site was recorded (0 cell, 1 cell, more than 1 cell, or debris). Reverse transcription and cDNA amplification was completed on each capture site of the C1 circuit. The amplified cDNA was then loaded into the Biomark qRT-PCR machine with pre-designed RGC-specific and RGC-subtype primers. Any values from a wells with zero or more than one cell were discarded in the analyses. The Ct values from the Biomark qRT-PCR analysis were sorted based on the expression of BRN3B and their expression levels were further constructed into heatmaps of specific RGC subtype classes using GraphPad Prism software.

Single-Cell RNA-Seq Data Collection and SRCCA Analysis

SRCCA was performed using published scRNA-seq datasets from day 70 hPSC retinal cultures (n = 3), and detailed methods for single-cell capture, cDNA preparation and library generation, sequencing, read mapping, quality control, and gene expression quantification were reported previously (Phillips et al., 2017). In

brief, cell capture and library preparations were performed with the Fluidigm C1 system according to manufacturer's instructions. cDNA was generated with the SMARTer PCR cDNA Synthesis kit (Clontech) and amplified with the Advantage 2 PCR kit (Clontech). Single-cell cDNA libraries were fragmented and amplified with the Nextera XT DNA sample preparation and index kit (Illumina), multiplexed (24–48 libraries per lane), and 51-bp single-end reads were sequenced (Illumina HiSeq 2500 System). FASTQ files were generated by CASAVA (v.1.8.2) and mapped using Bowtie (v.0.12.8). RSEM (v.1.2.3) was used to calculate normalized gene expression values in transcripts per million. Quality control analysis was performed using the SinQC program (Jiang et al., 2016). SRCCA analysis of RGC target genes was performed by R programming language (http://www.R-project.org/). Correlating genes were ranked based on Spearman's correlation coefficients (rho) from high to low values. To determine overlap between SRCCA lists from multiple target genes, gene lists were imported into Venny 2.1 (http://bioinfogp.cnb.csic.es/tools/venny/) or Meta-Chart Venn diagram generator (https://www.meta-chart.com/venn#/display). Single cell RNA-seq reads were submitted to GEO: GSE98556.

SUPPLEMENTAL INFORMATION

Supplemental Information includes two figures and two tables and can be found with this article online at https://doi.org/10.1016/j.stemcr.2018.02.010.

AUTHOR CONTRIBUTIONS

K.B.L., experimental design, data collection and interpretation, and manuscript writing. S.K.O., experimental design. C.M.F., data collection. M.J.P., data collection and interpretation and manuscript writing. P.J., data collection and interpretation. D.M.G., experimental design and data interpretation. J.S.M., experimental design, data interpretation, manuscript writing, and final approval of manuscript.

ACKNOWLEDGMENTS

We would like to thank Andrea Gunawan from the Flow Cytometry Core at IUPUI for her assistance in running the single-cell C1 and qRT-PCR assays. Grant support was provided by the National Eye Institute (R01 EY024984 to J.S.M.; R01 EY21218 to D.M.G.), the Indiana Department of Health Brain and Spinal Cord Injury Fund (to J.S.M.), an IU Collaborative Research Grant from the Office of the Vice President for Research (to J.S.M.), an award from the IU Signature Center for Brain and Spinal Cord Injury (to J.S.M.), an IUPUI Graduate Office First Year University Fellowship (to K.B.L.), and the Purdue Research Foundation Fellowship (to K.B.L.). This publication was also supported by the Stark Neuroscience Research Institute/Eli Lilly and Company predoctoral fellowship (to S.K.O.) and an Indiana Clinical and Translational Sciences predoctoral fellowship (to S.K.O.), funded in part by grant no. UL1TR001108 from the NIH, National Center for Advancing Translational Sciences.

Received: December 1, 2017
Revised: February 20, 2018
Accepted: February 21, 2018
Published: March 22, 2018

REFERENCES

Berson, D.M. (2008). Retinal ganglion cell types and their central projections. In The Senses: A Comprehensive Reference, Vol. 1, R.H. Masland and T.D. Albright, eds. (Elsevier), pp. 491–519.

Chew, K.S., Renna, J.M., McNeill, D.S., Fernandez, D.C., Keenan, W.T., Thomsen, M.B., Ecker, J.L., Loevinsohn, G.S., VanDunk, C., Vicarel, D.C., et al. (2017). A subset of ipRGCs regulates both maturation of the circadian clock and segregation of retinogeniculate projections in mice. Elife *6*. https://doi.org/10.7554/eLife.22861.

Croner, L.J., and Kaplan, E. (1995). Receptive fields of P and M ganglion cells across the primate retina. Vision Res. *35*, 7–24.

Cruz-Martín, A., El-Danaf, R.N., Osakada, F., Sriram, B., Dhande, O.S., Nguyen, P.L., Callaway, E.M., Ghosh, A., and Huberman, A.D. (2014). A dedicated circuit links direction-selective retinal ganglion cells to the primary visual cortex. Nature *507*, 358–361.

De la Huerta, I., Kim, I.J., Voinescu, P.E., and Sanes, J.R. (2012). Direction-selective retinal ganglion cells arise from molecularly specified multipotential progenitors. Proc. Natl. Acad. Sci. USA *109*, 17663–17668.

Dhande, O.S., Stafford, B.K., Lim, J.A., and Huberman, A.D. (2015). Contributions of retinal ganglion cells to subcortical visual processing and behaviors. Annu. Rev. Vis. Sci. *1*, 291–328.

Duan, X., Qiao, M., Bei, F., Kim, I.J., He, Z., and Sanes, J.R. (2015). Subtype-specific regeneration of retinal ganglion cells following axotomy: effects of osteopontin and mTOR signaling. Neuron *85*, 1244–1256.

El-Danaf, R.N., and Huberman, A.D. (2015). Characteristic patterns of dendritic remodeling in early-stage glaucoma: evidence from genetically identified retinal ganglion cell types. J. Neurosci. *35*, 2329–2343.

Gill, K.P., Hung, S.S., Sharov, A., Lo, C.Y., Needham, K., Lidgerwood, G.E., Jackson, S., Crombie, D.E., Nayagam, B.A., Cook, A.L., et al. (2016). Enriched retinal ganglion cells derived from human embryonic stem cells. Sci. Rep. *6*, 30552.

Gleeson, J.G., Lin, P.T., Flanagan, L.A., and Walsh, C.A. (1999). Doublecortin is a microtubule-associated protein and is expressed widely by migrating neurons. Neuron *23*, 257–271.

Goodman, K.M., Yamagata, M., Jin, X., Mannepalli, S., Katsamba, P.S., Ahlsén, G., Sergeeva, A.P., Honig, B., Sanes, J.R., and Shapiro, L. (2016). Molecular basis of sidekick-mediated cell-cell adhesion and specificity. Elife *5*. https://doi.org/10.7554/eLife.19058.

Hattar, S., Lucas, R.J., Mrosovsky, N., Thompson, S., Douglas, R.H., Hankins, M.W., Lem, J., Biel, M., Hofmann, F., Foster, R.G., et al. (2003). Melanopsin and rod-cone photoreceptive systems account for all major accessory visual functions in mice. Nature *424*, 76–81.

Hirami, Y., Osakada, F., Takahashi, K., Okita, K., Yamanaka, S., Ikeda, H., Yoshimura, N., and Takahashi, M. (2009). Generation of retinal cells from mouse and human induced pluripotent stem cells. Neurosci. Lett. *458*, 126–131.

Huberman, A.D., Manu, M., Koch, S.M., Susman, M.W., Lutz, A.B., Ullian, E.M., Baccus, S.A., and Barres, B.A. (2008). Architecture and activity-mediated refinement of axonal projections from a mosaic of genetically identified retinal ganglion cells. Neuron *59*, 425–438.

Huberman, A.D., Wei, W., Elstrott, J., Stafford, B.K., Feller, M.B., and Barres, B.A. (2009). Genetic identification of an on-off direction-selective retinal ganglion cell subtype reveals a layer-specific subcortical map of posterior motion. Neuron *62*, 327–334.

Jacoby, J., and Schwartz, G.W. (2017). Three small-receptive-field ganglion cells in the mouse retina are distinctly tuned to size, speed, and object motion. J. Neurosci. *37*, 610–625.

Jiang, P., Thomson, J.A., and Stewart, R. (2016). Quality control of single-cell RNA-seq by SinQC. Bioinformatics *32*, 2514–2516.

Kaplan, E., and Benardete, E. (2001). The dynamics of primate retinal ganglion cells. Prog. Brain Res. *134*, 17–34.

Kay, J.N., De la Huerta, I., Kim, I.J., Zhang, Y., Yamagata, M., Chu, M.W., Meister, M., and Sanes, J.R. (2011). Retinal ganglion cells with distinct directional preferences differ in molecular identity, structure, and central projections. J. Neurosci. *31*, 7753–7762.

Keller, G. (2005). Embryonic stem cell differentiation: emergence of a new era in biology and medicine. Genes Dev. *19*, 1129–1155.

Kim, I.J., Zhang, Y., Yamagata, M., Meister, M., and Sanes, J.R. (2008). Molecular identification of a retinal cell type that responds to upward motion. Nature *452*, 478–482.

Kim, T.J., and Jeon, C.J. (2006). Morphological classification of parvalbumin-containing retinal ganglion cells in mouse: single-cell injection after immunocytochemistry. Invest. Ophthalmol. Vis. Sci. *47*, 2757–2764.

Krieger, B., Qiao, M., Rousso, D.L., Sanes, J.R., and Meister, M. (2017). Four alpha ganglion cell types in mouse retina: function, structure, and molecular signatures. PLoS One *12*, e0180091.

Krishnaswamy, A., Yamagata, M., Duan, X., Hong, Y.K., and Sanes, J.R. (2015). Sidekick 2 directs formation of a retinal circuit that detects differential motion. Nature *524*, 466–470.

Lamba, D.A., Karl, M.O., Ware, C.B., and Reh, T.A. (2006). Efficient generation of retinal progenitor cells from human embryonic stem cells. Proc. Natl. Acad. Sci. USA *103*, 12769–12774.

Leventhal, A.G., Rodieck, R.W., and Dreher, B. (1981). Retinal ganglion cell classes in the Old World monkey: morphology and central projections. Science *213*, 1139–1142.

Levin, L.A. (2005). Retinal ganglion cells and supporting elements in culture. J. Glaucoma *14*, 305–307.

Majander, A., João, C., Rider, A.T., Henning, G.B., Votruba, M., Moore, A.T., Yu-Wai-Man, P., and Stockman, A. (2017). The pattern of retinal ganglion cell loss in OPA1-related autosomal dominant optic atrophy inferred from temporal, spatial, and chromatic sensitivity losses. Invest. Ophthalmol. Vis. Sci. *58*, 502–516.

Meyer, J.S., Howden, S.E., Wallace, K.A., Verhoeven, A.D., Wright, L.S., Capowski, E.E., Pinilla, I., Martin, J.M., Tian, S., Stewart, R., et al. (2011). Optic vesicle-like structures derived from human pluripotent stem cells facilitate a customized approach to retinal disease treatment. Stem Cells *29*, 1206–1218.

Meyer, J.S., Shearer, R.L., Capowski, E.E., Wright, L.S., Wallace, K.A., McMillan, E.L., Zhang, S.C., and Gamm, D.M. (2009). Modeling early retinal development with human embryonic and induced pluripotent stem cells. Proc. Natl. Acad. Sci. USA *106*, 16698–16703.

Ohlemacher, S.K., Iglesias, C.L., Sridhar, A., Gamm, D.M., and Meyer, J.S. (2015). Generation of highly enriched populations of optic vesicle-like retinal cells from human pluripotent stem cells. Curr. Protoc. Stem Cell Biol. *32*, 1H.8.1–20.

Ohlemacher, S.K., Sridhar, A., Xiao, Y., Hochstetler, A.E., Sarfarazi, M., Cummins, T.R., and Meyer, J.S. (2016). Stepwise differentiation of retinal ganglion cells from human pluripotent stem cells enables analysis of glaucomatous neurodegeneration. Stem Cells *34*, 1553–1562.

Osakada, F., Jin, Z.B., Hirami, Y., Ikeda, H., Danjyo, T., Watanabe, K., Sasai, Y., and Takahashi, M. (2009). In vitro differentiation of retinal cells from human pluripotent stem cells by small-molecule induction. J. Cell Sci. *122*, 3169–3179.

Panda, S., Sato, T.K., Castrucci, A.M., Rollag, M.D., DeGrip, W.J., Hogenesch, J.B., Provencio, I., and Kay, S.A. (2002). Melanopsin (Opn4) requirement for normal light-induced circadian phase shifting. Science *298*, 2213–2216.

Pang, J.J., Gao, F., and Wu, S.M. (2003). Light-evoked excitatory and inhibitory synaptic inputs to ON and OFF alpha ganglion cells in the mouse retina. J. Neurosci. *23*, 6063–6073.

Phillips, M.J., Jiang, P., Howden, S., Barney, P., Min, J., York, N.W., Chu, L.F., Capowski, E.E., Cash, A., Jain, S., et al. (2017). A novel approach to single cell RNA-sequence analysis facilitates in silico gene reporting of human pluripotent stem cell-derived retinal cell types. Stem Cells *36*, 313–324.

Puyang, Z., Gong, H.Q., He, S.G., Troy, J.B., Liu, X., and Liang, P.J. (2017). Different functional susceptibilities of mouse retinal ganglion cell subtypes to optic nerve crush injury. Exp. Eye Res. *162*, 97–103.

Rachel, R.A., Dolen, G., Hayes, N.L., Lu, A., Erskine, L., Nowakowski, R.S., and Mason, C.A. (2002). Spatiotemporal features of early neuronogenesis differ in wild-type and albino mouse retina. J. Neurosci. *22*, 4249–4263.

Riazifar, H., Jia, Y., Chen, J., Lynch, G., and Huang, T. (2014). Chemically induced specification of retinal ganglion cells from human embryonic and induced pluripotent stem cells. Stem Cells Transl. Med. *3*, 424–432.

Rokicki, W., Dorecka, M., and Romaniuk, W. (2007). Retinal ganglion cells death in glaucoma – mechanism and potential treatment. Part II. Klin. Oczna *109*, 353–355.

Rousso, D.L., Qiao, M., Kagan, R.D., Yamagata, M., Palmiter, R.D., and Sanes, J.R. (2016). Two pairs of ON and OFF retinal ganglion cells are defined by intersectional patterns of transcription factor expression. Cell Rep. *15*, 1930–1944.

Sanes, J.R., and Masland, R.H. (2015). The types of retinal ganglion cells: current status and implications for neuronal classification. Annu. Rev. Neurosci. *38*, 221–246.

Schmidt, T.M., Chen, S.K., and Hattar, S. (2011). Intrinsically photosensitive retinal ganglion cells: many subtypes, diverse functions. Trends Neurosci. *34*, 572–580.

Siegert, S., Cabuy, E., Scherf, B.G., Kohler, H., Panda, S., Le, Y.Z., Fehling, H.J., Gaidatzis, D., Stadler, M.B., and Roska, B. (2012). Transcriptional code and disease map for adult retinal cell types. Nat. Neurosci. *15*, 487–495, s481-482.

Sluch, V.M., Davis, C.H., Ranganathan, V., Kerr, J.M., Krick, K., Martin, R., Berlinicke, C.A., Marsh-Armstrong, N., Diamond, J.S., Mao, H.Q., et al. (2015). Differentiation of human ESCs to retinal ganglion cells using a CRISPR engineered reporter cell line. Sci. Rep. *5*, 16595.

Sridhar, A., Ohlemacher, S.K., Langer, K.B., and Meyer, J.S. (2016). Robust differentiation of mRNA-reprogrammed human induced pluripotent stem cells toward a retinal lineage. Stem Cells Transl. Med. *5*, 417–426.

Sridhar, A., Steward, M.M., and Meyer, J.S. (2013). Nonxenogeneic growth and retinal differentiation of human induced pluripotent stem cells. Stem Cells Transl. Med. *2*, 255–264.

Struebing, F.L., Lee, R.K., Williams, R.W., and Geisert, E.E. (2016). Genetic networks in mouse retinal ganglion cells. Front. Genet. *7*, 169.

Sümbül, U., Song, S., McCulloch, K., Becker, M., Lin, B., Sanes, J.R., Masland, R.H., and Seung, H.S. (2014). A genetic and computational approach to structurally classify neuronal types. Nat. Commun. *5*, 3512.

Sweeney, N.T., James, K.N., Nistorica, A., Lorig-Roach, R.M., and Feldheim, D.A. (2017). Expression of transcription factors divides retinal ganglion cells into distinct classes. J. Comp. Neurol. https://doi.org/10.1002/cne.24172.

Sweeney, N.T., Tierney, H., and Feldheim, D.A. (2014). Tbr2 is required to generate a neural circuit mediating the pupillary light reflex. J. Neurosci. *34*, 5447–5453.

Takahashi, K., Okita, K., Nakagawa, M., and Yamanaka, S. (2007). Induction of pluripotent stem cells from fibroblast cultures. Nat. Protoc. *2*, 3081–3089.

Tanaka, T., Yokoi, T., Tamalu, F., Watanabe, S., Nishina, S., and Azuma, N. (2016). Generation of retinal ganglion cells with functional axons from mouse embryonic stem cells and induced pluripotent stem cells. Invest. Ophthalmol. Vis. Sci. *57*, 3348–3359.

Teotia, P., Chopra, D.A., Dravid, S.M., Van Hook, M.J., Qiu, F., Morrison, J., Rizzino, A., and Ahmad, I. (2017). Generation of functional human retinal ganglion cells with target specificity from pluripotent stem cells by chemically defined recapitulation of developmental mechanism. Stem Cells *35*, 572–585.

Thomson, J.A., Itskovitz-Eldor, J., Shapiro, S.S., Waknitz, M.A., Swiergiel, J.J., Marshall, V.S., and Jones, J.M. (1998). Embryonic stem cell lines derived from human blastocysts. Science *282*, 1145–1147.

Trimarchi, J.M., Stadler, M.B., and Cepko, C.L. (2008). Individual retinal progenitor cells display extensive heterogeneity of gene expression. PLoS One *3*, e1588.

Trimarchi, J.M., Stadler, M.B., Roska, B., Billings, N., Sun, B., Bartch, B., and Cepko, C.L. (2007). Molecular heterogeneity of developing retinal ganglion and amacrine cells revealed through single cell gene expression profiling. J. Comp. Neurol. *502*, 1047–1065.

Triplett, J.W., Wei, W., Gonzalez, C., Sweeney, N.T., Huberman, A.D., Feller, M.B., and Feldheim, D.A. (2014). Dendritic and axonal targeting patterns of a genetically-specified class of retinal ganglion cells that participate in image-forming circuits. Neural Dev. *9*, 2.

Trost, A., Schroedl, F., Marschallinger, J., Rivera, F.J., Bogner, B., Runge, C., Couillard-Despres, S., Aigner, L., and Reitsamer, H.A. (2014). Characterization of dsRed2-positive cells in the doublecortin-dsRed2 transgenic adult rat retina. Histochem. Cell Biol. *142*, 601–617.

Wei, W., and Feller, M.B. (2011). Organization and development of direction-selective circuits in the retina. Trends Neurosci. *34*, 638–645.

Yonehara, K., Shintani, T., Suzuki, R., Sakuta, H., Takeuchi, Y., Nakamura-Yonehara, K., and Noda, M. (2008). Expression of SPIG1 reveals development of a retinal ganglion cell subtype projecting to the medial terminal nucleus in the mouse. PLoS One *3*, e1533.

Yu, J., Vodyanik, M.A., Smuga-Otto, K., Antosiewicz-Bourget, J., Frane, J.L., Tian, S., Nie, J., Jonsdottir, G.A., Ruotti, V., Stewart, R., et al. (2007). Induced pluripotent stem cell lines derived from human somatic cells. Science *318*, 1917–1920.

Zhong, X., Gutierrez, C., Xue, T., Hampton, C., Vergara, M.N., Cao, L.H., Peters, A., Park, T.S., Zambidis, E.T., Meyer, J.S., et al. (2014). Generation of three-dimensional retinal tissue with functional photoreceptors from human iPSCs. Nat. Commun. *5*, 4047.

Stem Cell Reports, Volume *10*

Supplemental Information

Retinal Ganglion Cell Diversity and Subtype Specification from Human Pluripotent Stem Cells

Kirstin B. Langer, Sarah K. Ohlemacher, M. Joseph Phillips, Clarisse M. Fligor, Peng Jiang, David M. Gamm, and Jason S. Meyer

Table S1: Major RGC Subtype Classes, their molecular makers, and their function

Retinal Ganglion Cell Subtype	Molecular Markers	Function
ON-OFF Direction Selective-RGCs	CART, CDH6, MMP17, DRD4, HB9, THRH, BD	Respond to preferred directional motion of bright and dark stimuli
ON Direction Selective-RGCs	HOXD10, FSTL4	Respond to preferred directional motion of bright stimulus
Alpha-RGCs	SPP1, KCNG4, CB2, SMI32	Differentially respond to bright and dark stimuli
Intrinsically photosensitive-RGCs	Melanopsin, TBR2	Respond to light to regulate circadian rhythms and pupillary reflex
W3B-RGCs	SDK2, TYW3	Respond to stimuli that fall within the center of the receptive field.
J-RGCs	JAMB	Respond to directional movement of stimuli from soma-to-dendrite
PV-RGCs	PVALB	Transient responses with small receptive fields and strong surrounds.

Table S1: This table indicates the major classes of RGC subtypes, previously identified molecular markers for each class, and their physiological function in the visual transmission. Related to Figures 3-7.

**Table S2**: Primary Antibody Information			
Primary Antibodies	Company	Catalog Number	Dilution
BRN3	Santa Cruz	SC-15375	1:200
βIII-Tubulin	Covance	PRB-435P/802001	1:1000
CART	Phoenix Pharmaceuticals	H-003-62	1:100
CB2	R&D Systems	MAB3655	1:50
CDH6	Sigma-Aldrich	HPA007047	1:100
DCX	Santa Cruz	SC-271390	1:50
FSTL4	Novus	91913	1:200
HUC/D	Molecular Probes	A-21271	1:200
ISLET1	DSHB	40.2D6	1:200
MAP2	Santa Cruz	SC-20172	1:200
MELANOPSIN	ThermoScientific	PA1-781	1:500
SMI-32	Calbiochem Millipore	NE1023	1:500
SPP1	Abcam	Ab91655	1:50

Table S2: Antibodies used in this study, related to Figures 1, 3, 4, 5, and 7.

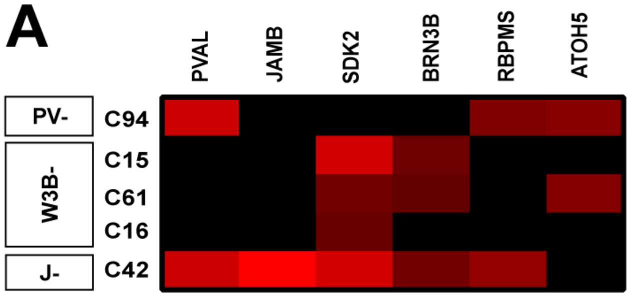

Figure S1: **Single cell qRT-PCR reveals expression of markers for other RGC subtypes.** A variety of other subtypes were characterized using single cell qRT-PCR. (A) PV-, W3B-, and J-RGCs were identified through the expression of PVALB, SDK2, and JAMB respectively, in addition to their combinatorial expression with a variety of other RGC-associated markers.

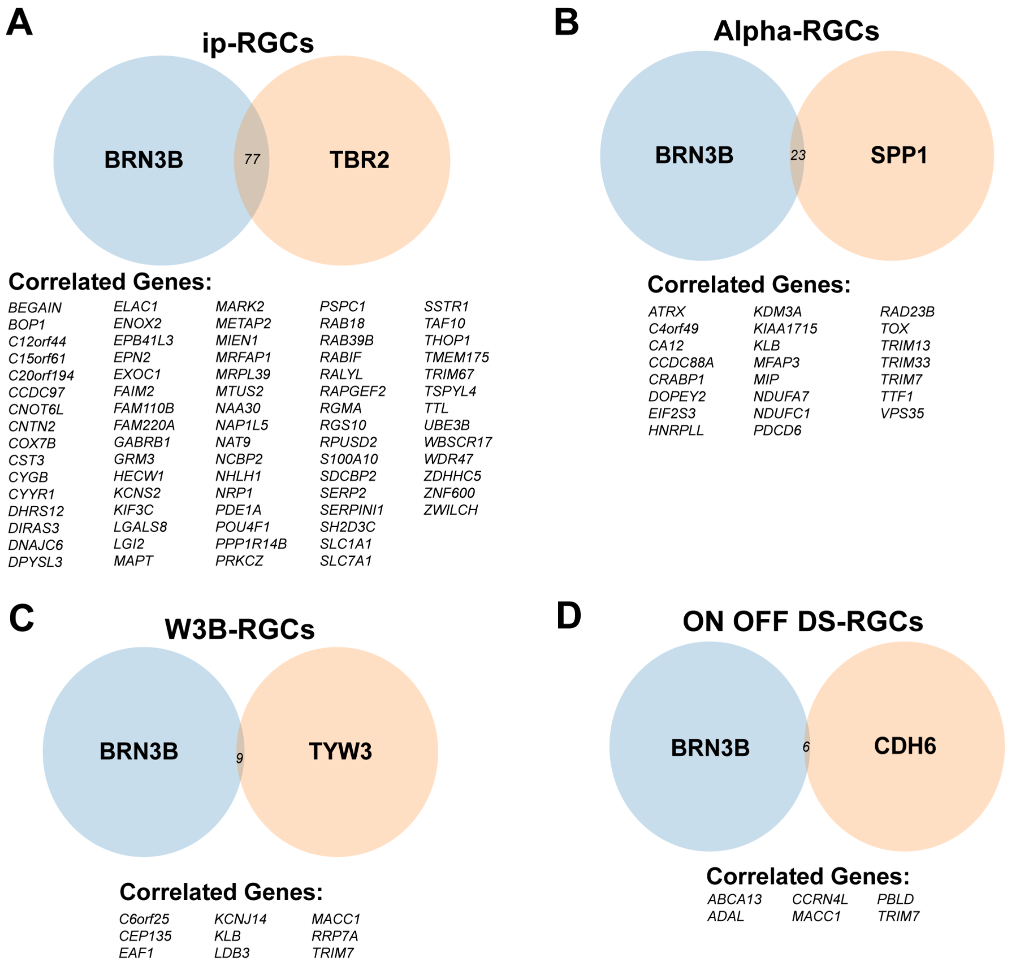

Figure S2: Single cell RNA-seq analyses indicate correlative genes for major RGC subtype classes. Single cell RNAseq analyses of numerous subtypes reveals closely correlated genes. (A-D) SRCCA for BRN3B using the top 1000 correlating genes was combined with SRCCA for subtype specific markers and revealed correlated genes. For ip-, Alpha-, W3B-, and ON OFF DS-RGCs 77, 23, 9, and 6 genes were found closely correlated within the top 1000 genes.
